# Non-Destructive Testing of Carbon Fibre Reinforced Plastics (CFRP) Using a Dual Transmitter-Receiver Differential Eddy Current Test Probe

**DOI:** 10.3390/s22186761

**Published:** 2022-09-07

**Authors:** Ronghua Zhang, Junyu Wang, Shiyu Liu, Ming Ma, Hongying Fang, Junhua Cheng, Danqi Zhang

**Affiliations:** 1 School of Artificial Intelligence, Tiangong University, Tianjin 300387, China; 2 School of Control Science and Engineering, Tiangong University, Tianjin 300387, China; 3 School of Life Sciences, Tiangong University, Tianjin 300387, China

**Keywords:** eddy-current test, electrical anisotropy, detection sensitivity, feature extraction

## Abstract

Transmitter-receiver (T-R) probes are widely used in the eddy-current testing of carbon fibre reinforced plastics (CFRP). However, T-R probes have the disadvantage of being highly sensitive to lift-off. On this basis, lift-off interference can be eliminated by differential structure. However, due to the electrical anisotropy of CFRP, the detection sensitivity of the side-by-side T-R probe and traditional R-T-R differential probe are greatly affected by the scanning angle, and the probe often needs to scan the sample along a specific path to achieve the ideal required detection effect. To solve these problems, a symmetrical dual-transmit-dual-receive (TR-TR) differential probe is designed in this paper. The detection performance of the TR-TR probe was verified by simulation and experiments. Results show that the TR-TR probe is less affected by the scanning angle and lift-off when used in CFRP defect detection, and has high detection sensitivity. However, the imaging results of the TR-TR probe do not show the defect characteristics straightforwardly. To solve this problem, a defect feature extraction algorithm is proposed in this paper. The results show that the defect feature extraction algorithm can locate and size the defect more accurately and improve the signal-to-noise ratio.

## 1. Introduction

As an emerging structural functional material, carbon fibre reinforced plastics (CFRPs) have already been widely used in aerospace [1], sports equipment, and automotive manufacturing. At the same time, the demand for nondestructive testing of CFRP is growing. At present, methods such as ultrasonic testing [2], X-ray [3], infrared thermography [4], and eddy-current testing [5] can be used for nondestructive testing of CFRP. Among them, the eddy-current testing method is widely used in CFRP surface and near-surface defect detection due to its advantages of having a fast response and being noncontact. The presence of defect(s) can be detected based on changes in the voltage induced on the receiver coil [6]. However, the low conductivity and electrical anisotropy of CFRP [7] poses difficulty for small defect detection. The performance of eddy-current nondestructive testing largely depends on the probe structure, so it is of great significance to design suitable probes according to the electrical characteristics of CFRP.

Due to the low conductivity of CFRP, high excitation frequency is typically used to achieve higher sensitivity. R. Grimberg et al., designed an orthogonal T-R probe to image the fibre arrangement of CFRP [8]. Gerhard Mook et al., used a high-frequency excited T-R probe to measure the carbon fibre arrangement direction and local defects in CFRP [9]. Koichi Mizukami et al., designed a rectangular probe for the detection of carbon fibre waviness. The probe can detect fibre bending with the high excitation frequency and can determine the position of fibre deformation by complex plane analysis [10]. They also designed another probe to detect 10 mm × 10 mm delamination defects embedded in the CFRP of braided structures [11]. Dehui Wu et al. proposed a figure-8 transmitting coil to detect the degree of fibre bending [12]. A probe based on a microprinted circuit board is designed to detect CFRP defects [13]. Dario J. Pasadas et al., proposed a dual excitation differential probe and enhanced excitation using resonance frequency techniques and detected defects in CFRP at an excitation frequency of 1 MHz [14]. Carsten Schmidt et al., detected fibre overlaps and gaps formed during the preparation of CFRP under high-frequency excitation [15]. Koyama Kiyoshi et al. designed an orthogonal rectangular probe to enhance the distribution of eddy-currents along the fibre direction, realizing the detection of unidirectional CFRP and quasi-isotropic CFRP defects [16]. However, high-frequency excitation leads to serious signal transmission interference, imposing higher instrument specifications. Other researchers seek to use low frequency excitation for defect detection. Jun Cheng et al. used a side-by-side T-R probe to detect CFRP under low-frequency excitation and successfully detected the location and size of different defects by scanning imaging [17]. In addition, the polar diagrams are depicted to characterize the anisotropy of CFRP laminate [18]. NaZhang et al., proposes a flexible eddy-current testing (ECT) probe, the probe can be bent to fit a curved surface, and a small defect located on the curved surface of a CFRP sample can be detected by the probe [19].

In this paper, we design a dual excitation dual receiver (TR-TR) differential probe and use it for CFRP defect detection. The TR-TR differential probe has high detection sensitivity. The differential structure can effectively suppress common-mode interference caused by lift-off and primary magnetic field. The transmitter coil and the receiver coil are coaxial, which ensures that the detection signal of the receiver coil is not affected by the rotation of the probe. In addition, in view of the problem that the TR-TR differential probe cannot accurately reflect the defect features, this paper proposes a defect feature extraction algorithm. The results show that the algorithm can improve the accuracy of defect localization and increase the contrast between the crack and the surrounding area. In addition, the algorithm can improve the spatial resolution of the probe to extract braided voids in braided CFRP.

## 2. Probe Design

The sensitivity of the probe to eddy-current changes depends on its structure. It is of great significance to improve the probe structure according to the eddy-current distribution characteristics of CFRP. In this paper, according to Maxwell’s equations shown in Equation (1), the finite element simulation method is used to analyse the eddy-current distribution characteristics of CFRP. On this basis, a probe suitable for CFRP eddy-current testing is designed.

(1) $$\begin{array}{l} \nabla \times \vec{H}=\vec{J}\\ \nabla \times \vec{E}=-\partial \vec{B}/\partial t\\ \nabla \times \vec{B}=0\\ \nabla \times \vec{D}=\rho \end{array}$$

where $\vec{H}$ is the magnetic field strength, $\vec{J}$ is the current density, $\vec{E}$ is the electric field strength, $\vec{B}$ is the magnetic flux density, D is the electric displacement vector, and ρ is free electron density.

**Figure 1:**
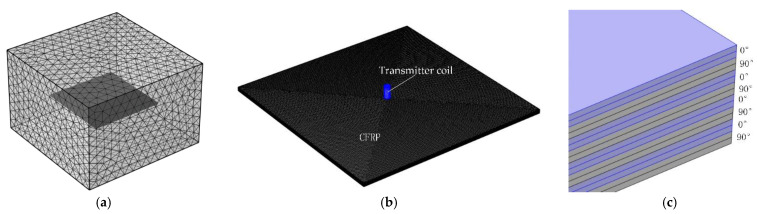
Simulation model: (**a**) air domain meshing; (**b**) meshing of coils and CFRP; (**c**) angle between fibre direction and X axis.

The parameters of the simulation model are as follows. In the practical use of CFRP, the method of multidirectional cross-woven is typically used to improve the strength of the material in different directions. This paper takes orthogonal CFRP as an example to conduct finite element analysis, and on this basis, designs a probe for CFRP defect detection. The values of electrical conductivity used in the simulations are typical values of electrical conductivity used for CFRP materials. In the simulation model, the electrical conductivity $\sigma_{l}$ along the fibre direction, $\sigma_{t}$ perpendicular to the fibre direction, and $\sigma_{cp}$ in the thickness direction of the sample are set to 10,000 S/m, 100 S/m, and 100 S/m, respectively [14]. The conductivity σ of each layer of CFRP can be represented by the third-order tensor matrix shown in Formula (2), where ∂ is the angle between the fibre direction and the x-axis. The CFRP model has a total of eight layers, the adjacent layers are orthogonal to each other. The eight layers have the same size; each layer is a cuboid of 100 mm × 100 mm × 0.25 mm. The eight layers are closely arranged to form a CFRP with a size of 100 mm × 100 mm × 2 mm. As shown in Figure 1c, set ∂ as [0°/90°/0°/90°/0°/90°/0°/90°]. In order to improve the simulation accuracy, each 0.25 mm thick layer is divided into two 0.125 mm thick sub-layers. The air domain is set as a cuboid of 180 mm × 180 mm × 120 mm. For the solver to converge, the air conductivity is set to 1 S/m, and this small nonzero value causes negligible error. In order to improve the solution accuracy of the simulation model, the coil and CFRP regions are divided by finer tetrahedral meshes, and the air domain is divided by fine tetrahedral meshes. All finite element simulations are based on the software COMSOL Multiphysics.

(2) $$\left[\sigma \right]=\left\{\begin{array}{ccc} \sigma_{l}\cos^{2}\partial +\sigma_{t}\sin^{2}\partial & \frac{\sigma_{l}-\sigma_{t}}{2}\sin2\partial & 0\\ \frac{\sigma_{l}-\sigma_{t}}{2}\sin2\partial & \sigma_{l}\sin^{2}\partial +\sigma_{t}\cos^{2}\partial & 0\\ 0 & 0 & \sigma_{cp} \end{array}\right\}$$

**Figure 2:**
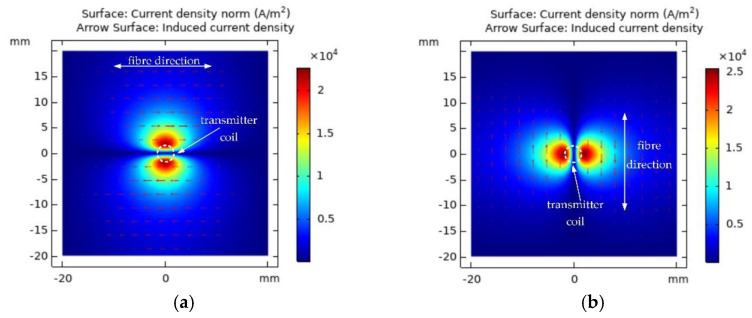
Eddy-current distribution (**a**) surface layer (∂ = 0°); (**b**) subsurface layer (∂ = 90°).

Circular coils have a highly axis-symmetrical geometry that produces a more concentrated magnetic field. This section will analyse the eddy-current distribution formed by the circular transmitter coil on CFRP. Transmitter coil parameter 1 is shown in Table 1 as Tx coil. Probe lift-off is set to 0.5 mm and low-frequency alternating current of 1 A at 100 kHz is applied to the transmitter coil. The eddy-current distribution in the same direction layer is similar, but the eddy-current density gradually decreases with the increase in depth [20]. Taking the surface layer (∂ = 0°) and subsurface layer (∂ = 90°) as an example, the top view of the eddy-current distribution in the surface layer and subsurface layer is obtained by simulation as shown in Figure 2. It can be seen from the figure that the eddy-current is mainly distributed along the fibre direction.

**Figure 3:**
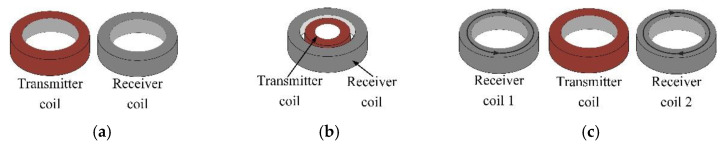
Probe structure: (**a**) traditional side by side T-R probe; (**b**) concentric T-R probe; (**c**) traditional R-T-R differential probe.

**Figure 4:**
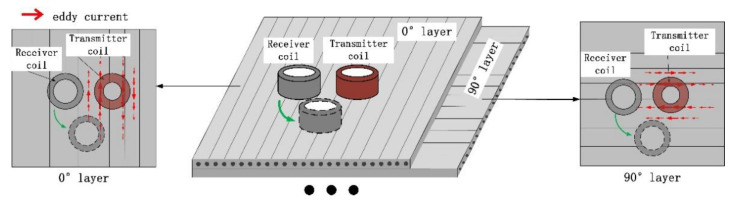
The effect of the orientation of the side-by-side T-R probe on its receiver coil measurement signal.

The side-by-side T-R probe shown in Figure 3a are widely used in eddy-current testing. The traditional T-R probe consists of transmitter coils and receiver coils side by side. Figure 4 shows schematic diagrams of the coupling of the eddy-current field and the receiver coil when the conventional T-R probes are placed at different angles. The green arrow in Figure 4 represents the direction of rotation of the T-R probe. As can be seen from the figure, there is a difference in the eddy-current density distribution around the transmitter coil. In CFRP laminate, the induced eddy-currents are stretched in the directions along the fibres and tightened in the directions perpendicular to the fibres. Therefore, the change in the angle between the probe direction (the direction of the centre line connecting the transmitter coil and the receiver coil) and the fibre direction will affect the strength of the output signal of the receiver coil.

In addition, the R-T-R differential probe shown in Figure 3c is also widely used in eddy-current testing. The R-T-R differential probe consists of a transmitter coil and two differentially connected receiver coils. Compared with the T-R probe, the R-T-R probe can reduce the interference of lift-off and primary magnetic field. According to the analysis in Figure 4, the R-T-R differential probe is similar to the side-by-side T-R probe, the detection sensitivity of the R-T-R differential probe is also affected by the direction of the probe. 

**Figure 5:**
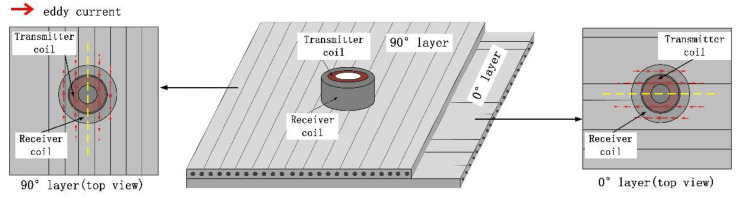
The effect of the orientation of the concentric T-R probe on its receiver coil measurement signal.

As shown in Figure 5, compared with the traditional side-by-side T-R probe, rotation of the concentric T-R probe does not change its electromagnetic coupling. Therefore, the detection sensitivity of the concentric T-R probe is not affected by the direction of the probe. 

**Figure 6:**
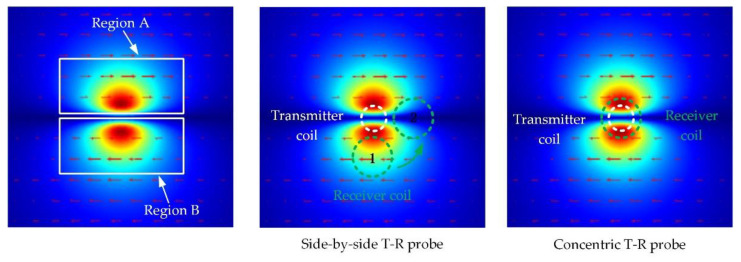
Comparison of detection sensitivity between side-by-side T-R probes and concentric T-R probes.

In addition, the concentric T-R probe has a higher detection sensitivity than the side-by-side probe. As shown in Figure 6, the surface layer is taken as an example. For the side-by-side T-R probe, when the receiver coil is placed at the position marked 1, the receiver coil is sensitive to eddy-current changes in region B, and eddy-current changes in region A have less effect on the receiver coil signal. When the receiver coil is located at the position marked 2, the eddy-current changes in Region A and Region B simultaneously affect the receiver coil signal. However, due to the lower eddy-current density at the location marked 2, the eddy-currents in Regions A and B have little effect on the receiver coil signal. For the concentric T-R probe, the receiver coil can sense the eddy-current changes in both areas A and B at the same time, and the eddy-current density in the area under the receiver coil of the concentric T-R probe is higher, and the degree of electromagnetic coupling between the receiver coil and eddy-current field is higher. Therefore, when the parameters of the coil are the same, the detection sensitivity of the concentric T-R probe is higher than that of the traditional T-R probe.

**Figure 7:**
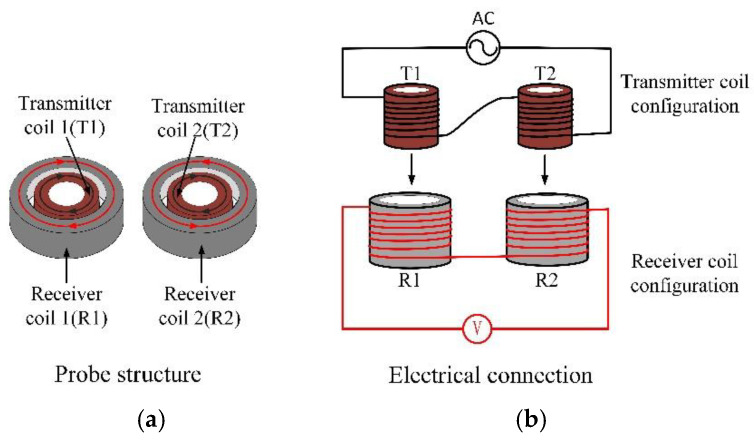
TR-TR differential probe: (**a**) probe structure; (**b**) coil connection method.

Based on this idea, this paper designed a novel differential probe whose structure is shown in Figure 7a. The new differential probe consists of two identical sets of concentrically nested transmitter coils and receiver coils. The two transmitter coils are connected in series in the same direction, and the receiver coil forms a differential structure. The specific wire connection method is shown in Figure 7b.

The TR-TR differential probe in this paper has the following advantages. Two transmitter coils are connected in series to generate a primary magnetic field in the same direction. When the probe is placed at a defect-free position, the receiving coil always induces an induced voltage of equal magnitude and opposite direction, and the total output voltage of the probe is 0. Therefore, the TR-TR probe is not affected by the primary magnetic field and lift-off, and has a higher signal-to-noise ratio. When the probe scans the defect position, the symmetry of the eddy-current distribution is broken, there is an induced voltage difference between the two receiver coils, and the output signal changes. At the same time, similar to the concentric T-R probe, the TR-TR differential probe has the advantage that the detection sensitivity is not affected by the scanning angle.

## 3. Verification of Probe Performance

This section uses finite element simulation to optimize the key parameters affecting the performance of the TR-TR probe, and then compares the detection sensitivity and anti-interference ability of the traditional side-by-side T-R probe, the concentric T-R probe, the traditional R-T-R differential probe and the TR-TR differential probe.

**Figure 8:**
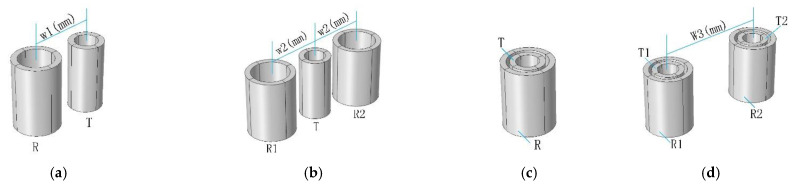
Probe Simulation Geometry. (**a**) Side−by−side T-R probe, (**b**) R-T-R differential probe, (**c**) concentric T-R probe, (**d**) TR-TR differential probe.

All four types of probes use the same transmitter coil and receiver coil, and the parameters of the transmitter coil and receiver coil are shown for Tx Coil and Rx Coil in Table 1, respectively. Figure 8 shows the established probe geometry model. In Figure 8, w1, w2 and w3 are the distances between the centre of the transmitter coil and the centre of the receiver coil of the T-R probe, the R-T-R differential probe, and the TR-TR differential probe, respectively.

**Figure 9:**
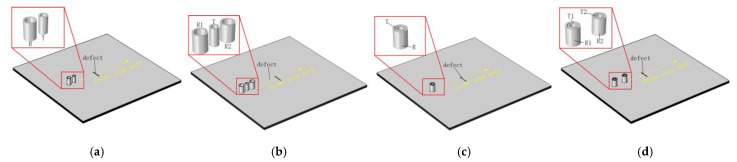
Schematic diagram of the simulation geometric model: (**a**) side−by−side T-R probe; (**b**) R-T-R differential probe; (**c**) concentric T-R probe; (**d**) TR-TR differential probe.

The setting of the CFRP conductivity value, the setting of the number and size of CFRP layers, the setting of the air domain size and the conductivity are the same as the parameters of the simulation model in Figure 1. In order to improve the solution accuracy of the simulation model, the coil and CFRP regions are divided by finer tetrahedral meshes, and the air domain is divided by fine tetrahedral meshes; on this basis, this section sets a defect with a size of 8 mm × 0.5 mm × 1 mm in the centre of the CFRP. Probe lift-off is set to 0.5 mm and alternating current of 1 A at 100 kHz is applied to the transmitter coil. Figure 9 is a schematic diagram of the simulation model after hiding the air domain.

### 3.1. Coil Distance

For T-R probes, R-T-R differential probes, and TR-TR differential probes, the distance between the coils (w1,w2 and w3) is an important factor affecting the detection sensitivity of the probe. This section compares the influence of the distance between the coils on the detection sensitivity of the probe by means of finite element simulation, and finally determines the values of the parameters w1, w2 and w3. The settings of the CFRP conductivity in the simulation model in this section are the same as those in the simulation model in Figure 1. The angle between the length of the defect and the positive direction of the x-axis is 90°. The probe scans the centre of the defect along the x-axis direction.

According to Table 1, the transmitter coil radius is 1.5 mm, and the receiver coil radius is 2.3 mm. Therefore, for the side-by-side T-R probe and the R-T-R differential probe, the minimum distance between the transmitter coil and the receiver coil (w1 or w2) is 3.8 mm. For TR-TR differential probe, the minimum distance between the transmitter coil and the receiver coil (w1 or w2) is 4.6 mm. 

**Figure 10:**
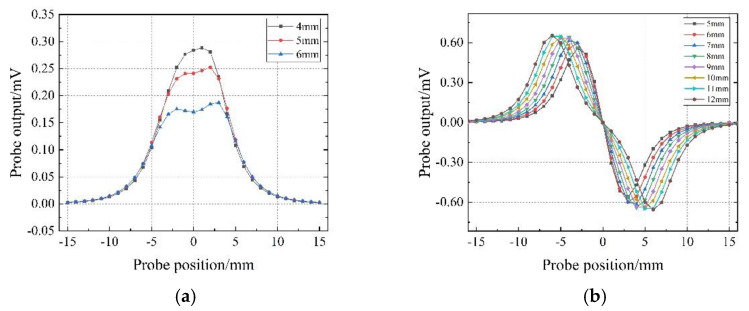
The output signal of the probe varies with the distance of the coil: (**a**) side−by−side T-R probe; (**b**) TR-TR differential probe.

The relationship between the detection sensitivity of the probe and the distance between the coils is shown in Figure 10. As shown in Figure 10a, for the T-R probe, the detection sensitivity of the probe decreases with the increase in w1. Due to the influence of the probe assembly accuracy in the subsequent experiments, the actual distance between the transmitter coil and the receiver coil in the experiment is 5 mm. In order to be consistent with the subsequent experiments, w1 is set to 5 mm in the simulation. For R-T-R differential probes, the relationship between detection sensitivity and coil distance is the same as for T-R probes, and w2 has the same settings as w1.

As shown in Figure 10b, the detection sensitivity of the TR-TR differential probe is proportional to the distance between the two groups of concentric coils (group1: T1 and R 1, group2: T2 and R2). However, when the distance between the two groups of concentric coils is greater than 10 mm, the increase in the distance between the two groups of coils cannot bring about a significant improvement in detection sensitivity. Considering the probe detection sensitivity and the compactness of the structure, the distance between the two groups of concentric coils (w3) is finally determined to be 10 mm. 

### 3.2. Scanning Angle

One of the advantages of the TR-TR differential probe is that the probe detection sensitivity is not affected by the scan angle. This section compares the detection sensitivity changes of the four probes in Figure 8 at different scanning angles.

Based on the simulation model in Figure 9, this section achieves the purpose of scanning defects from different directions by changing the fibre direction and the defect length direction. The specific settings are as follows.

In this section, the angle between the fibre direction and the positive direction of the x-axis is set to (0 + ∂)°/(90 + ∂)°/(0 + ∂)°/(90 + ∂)°/(0 + ∂)°/(90 + ∂)°/(0 + ∂)°/(90 + ∂)°. The angle between the length of the defect and the positive direction of the x-axis is (90 + ∂)°. The probe scans the defect along the x-axis direction. Among them, ∂ is a variable. Before the simulation calculation, the direction of fibres and defects can be adjusted by changing the value of ∂. In this way, the purpose of the probe to scan the defects from different angles is realized. 

**Figure 11:**
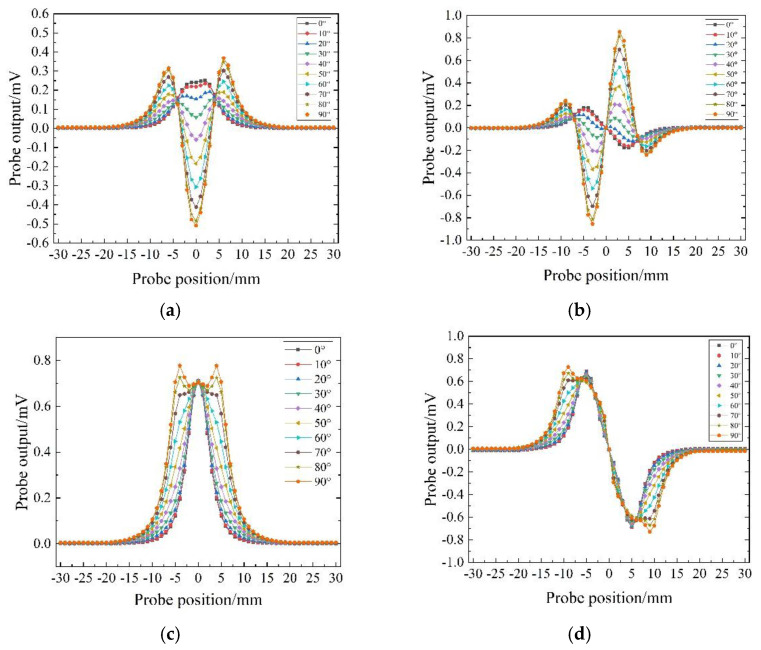
The output signal obtained by the probe scanning the defect in different directions: (**a**) side−by−side T-R probe; (**b**) R-T-R differential probe; (**c**) concentric T-R probe; (**d**) TR-TR differential probe.

The probe scans the CFRPs with defects and without defects along the same path and subtracts the two scanning results to obtain the output voltage change in the probe, as shown in Figure 11. The angles marked in Figure 11 are the values of ∂. 

The output voltage amplitude of the traditional T-R probe and the R-T-R differential probe varies greatly under different scanning angles, and the detection sensitivity is affected by the scanning angle. The output voltage amplitude of the TR-TR differential probe and the concentric T-R probe at different scanning angles are basically the same, and the detection sensitivity is not easily affected by the scanning angle; at most scanning angles, the detection sensitivity of the concentric T-R probe and the TR-TR differential probe is higher than the other two probes.

## 4. Experiments

**Figure 12:**
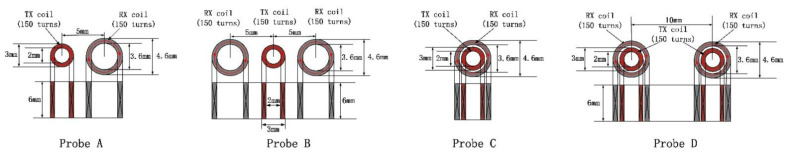
Structure and parameters of probes.

**Figure 13:**
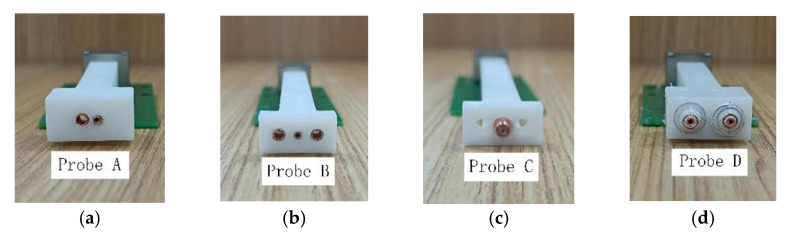
Physical image of probe: (**a**) Probe A, (**b**) Probe B, (**c**) Probe C, (**d**) Probe D.

To verify the performance of the designed probes, the probes were prepared for comparison experiments in this paper. The probe structure and parameters are shown in Figure 12. All probes use transmitter coils and receiver coils with the same parameters. In Figure 13, probe A is the traditional side-by-side T-R probe, probe B is the R-T-R differential probe, probe C is the concentric T-R probe, and probe D is the TR-TR differential probe.

**Figure 14:**
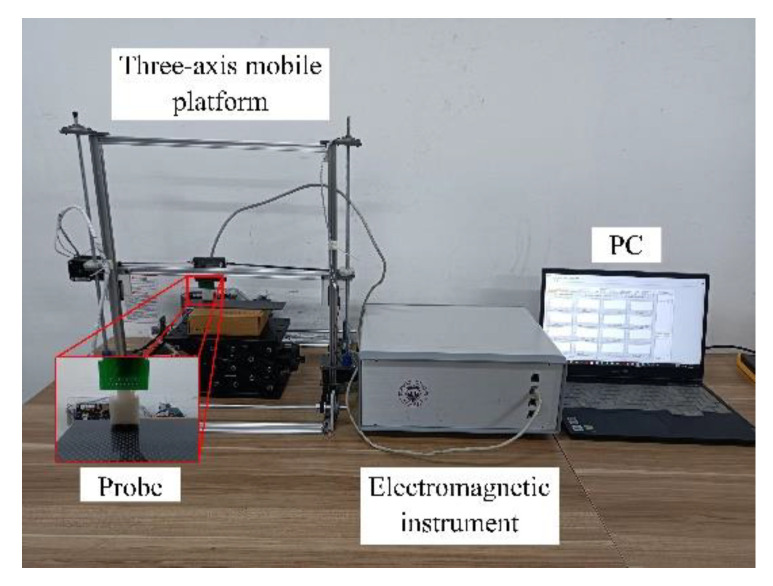
Experimental setup.

As shown in Figure 14, the experimental platform consists of a three-axis mobile platform, high-speed electromagnetic instrument, eddy-current probe, and PC host computer. The electromagnetic instrument, developed by the University of Manchester, features an advanced FPGA and can generate excitation signals from 1 to 200 kHz and perform digital demodulation at a rate of 100 kps to achieve high-speed processing.

**Figure 15:**
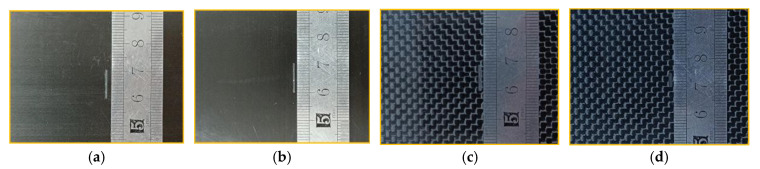
Photography of the sample: (**a**) sample 1, (**b**) sample 2, (**c**) sample 3, (**d**) sample 4.

In this experiment, the widely used orthogonal CFRP and braided CFRP were selected as the test objects. Defects are artificially created on the surface of the CFRP, and probes A and B are used to scan them and compare the measurement results. The prepreg of samples is P2255-17 (TORAY INDUSTRIES, Inc., Tokyo, Japan, layer thickness is 0.14 with TORAYCATM RESIN No. 2592). The carbon fibre content is 58 ± 5%. The thickness of all samples is about 2 mm, and the samples are shown in Figure 15. Samples 1 and 2 are composed of 14 fibre layers, and the adjacent layers are orthogonal to each other. The surface has a rectangular defect 10 mm long, 0.5 mm wide, and 0.8 mm deep. The defect length direction of sample 1 is perpendicular to the surface fibre direction, and the length direction of the defect of sample 2 is parallel to the surface fibre direction. Samples 3 and 4 consist of 11 orthogonal plies and two surface woven plies. The surface of sample 3 has a rectangular defect with a length of 9 mm, width of 0.5 mm, and depth of 0.8 mm. The surface of sample 4 has a rectangular defect with a length of 5 mm, width of 0.5 mm, and depth of 0.8 mm. The probe is fixed on the three-axis moving platform, with a lift-off of 1 mm. After comparison experiments, we found that when the excitation frequency is 100 kHz, the detection sensitivity of the probe is higher and the noise is small. Therefore, the final selected excitation frequency is 100 kHz.

### 4.1. Scanning Angle

**Figure 16:**
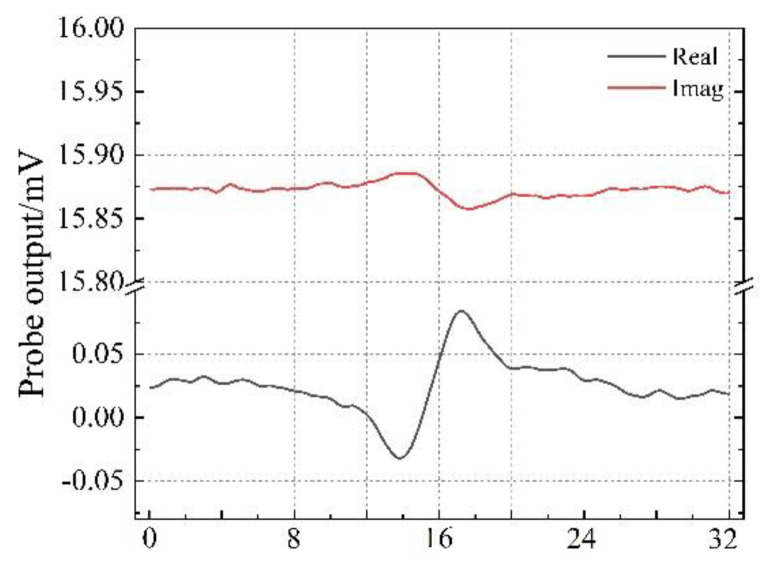
The real part and imaginary part of the probe output voltage signal.

This section take sample 1 as an example, compares the output voltage changes of the four probes in Figure 12 at different scanning angles. In the actual measurement process, it is found that the signal of the real part of the voltage is relatively obvious, while the signal of the imaginary part is weak. As shown in Figure 16, taking the TR-TR differential probe as an example, the variation in the real part of the output voltage is much larger than the variation in the imaginary part, and the imaginary part has a negligible effect on the measurement result. Therefore, the measurement signal we chose is the value of the real part of the voltage.

**Figure 17:**
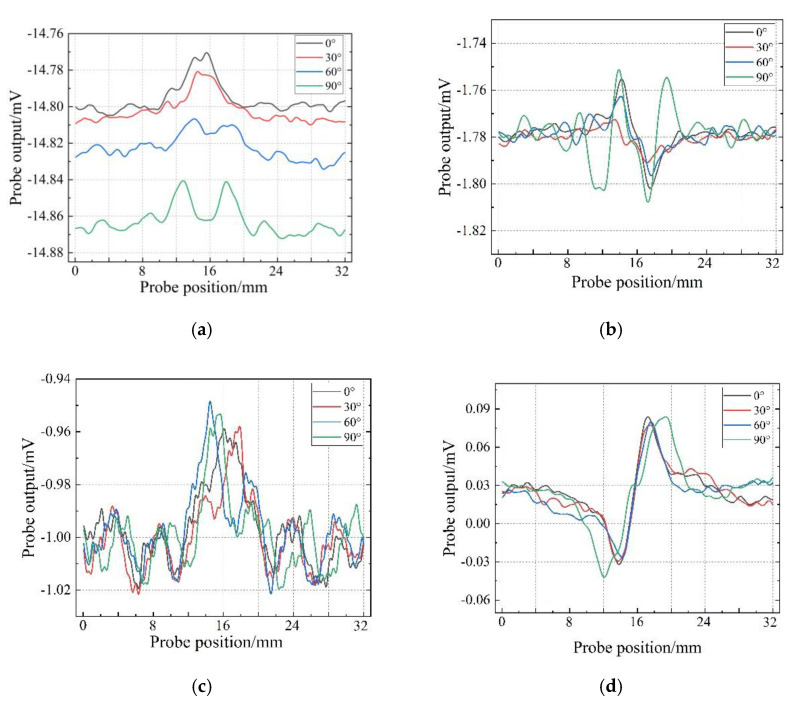
The output signal of the probe scanning the defect on the sample 1 in different directions: (**a**) side−by−side T-R probe; (**b**) R-T-R differential probe; (**c**) concentric T-R probe; (**d**) TR-TR differential probe.

The output voltages of the probes are shown in Figure 17. The angle marked in Figure 17 is the angle between the scanning path and the length direction of the defect. Figure 17 shows that the detection sensitivity of the traditional T-R probe and R-T-R differential probe is greatly affected by the scanning angle. The detection sensitivity of the probe is lower when the scanning angle is 30° and 60°. For the concentric T-R probe, the signal-to-noise ratio of the output signal of the probe is low due to the serious interference of the primary magnetic field on the induced voltage of the receiver coil. For the TR-TR differential probe, the scanning angle does not change the detection sensitivity of the probe, and the probe has strong anti-interference ability. It can also be seen from Figure 17 that the detection sensitivity of the TR-TR differential probe is higher than that of the traditional side-by-side probe and the traditional differential probe.

### 4.2. Imaging Experiments

**Figure 18:**
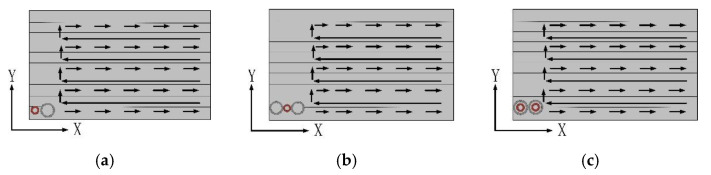
Scanning methods: (**a**) side-by-side T-R probe scanning method; (**b**) R-T-R differential probe scanning method; (**c**) TR-TR differential probe scanning method.

This section compares the difference in detection sensitivity and anti-interference ability between probes by means of imaging. It can be seen from Figure 17c that the signal-to-noise ratio of the output signal of the concentric T-R probe is low, which is not suitable for imaging. Therefore, this section only compares the imaging effects of the other three probes. As shown in Figure 18, the probe scans the sample in the direction perpendicular to the length of the defect in the C-scan mode. According to the calculation of the scanning speed and the sampling frequency of the equipment, the resolution of the x-axis is approximately 0.08 mm, and the resolution of the y-axis is 1 mm. 

**Figure 19:**
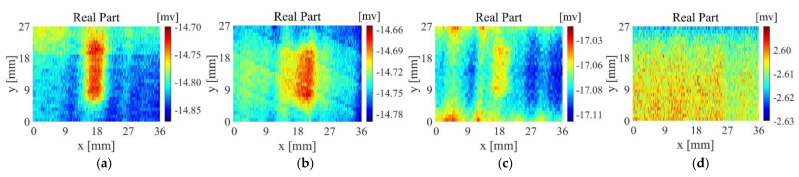
Scanning imaging of Probe A (**a**) sample 1, (**b**) sample 2, (**c**) sample 3, (**d**) sample 4.

**Figure 20:**
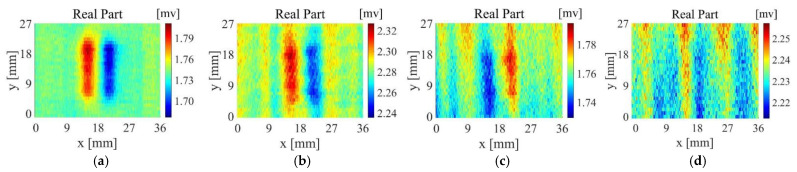
Scanning imaging of Probe B (**a**) sample 1, (**b**) sample 2, (**c**) sample 3, (**d**) sample 4.

**Figure 21:**
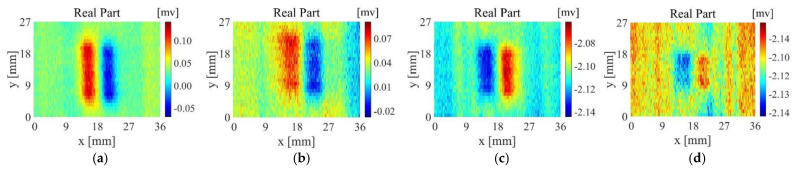
Scanning imaging of Probe D (**a**) sample 1, (**b**) sample 2, (**c**) sample 3, (**d**) sample 4.

Figure 19a–d are the imaging results of probe A scanning samples 1~4, Figure 20a–d are the imaging results of probe B scanning samples 1~4, and Figure 21a–d are the imaging results of probe D scanning samples 1~4. For Sample 1, all probes have good detection results, The output voltage of probe D has a larger change in amplitude. For Sample 2, the artefacts of the imaging results of probe A and probe B are more serious, and the defect imaging is blurred. Among them, probe B can observe the distribution of fibres from the background area. Probe D is affected by the fibre direction to a certain extent, but the artefacts in the defect area are relatively small, and the reflection of defect information is more accurate. For sample 3, probe A and probe B can detect the existence of defects but are seriously disturbed by noise. The scanning and imaging results of probe D can clearly observe the defect information. For sample 4, probe A and probe B cannot detect the defects at all, while the imaging results of probe B can still show defect information despite a higher background noise. 

The experimental results show that the TR-TR differential probe proposed in this paper has high detection sensitivity, and the ability to detect small defects is better. At the same time, the TR-TR differential probe has a strong anti-interference ability, and the measurement results are less affected by background noise such as lift-off.

## 5. Defect Feature Extraction Algorithms

**Figure 22:**
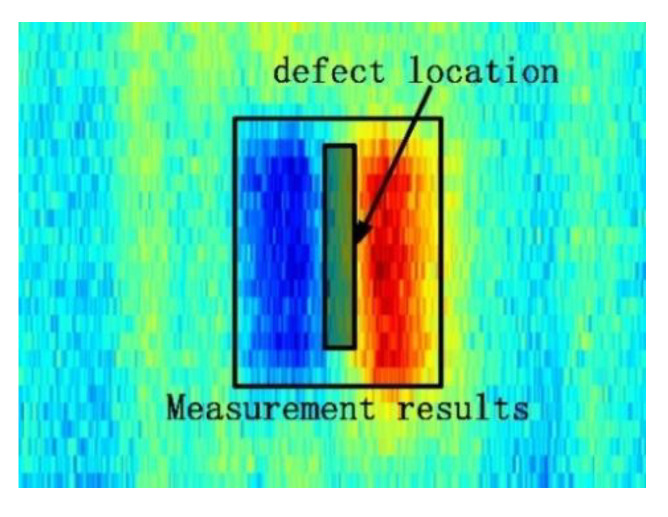
TR-TR differential probe imaging results and actual situation of defects.

Although the TR-TR differential probe has high detection sensitivity and strong anti-interference ability, its differential structure will cause the problem of inaccurate defect location. Figure 22 shows the imaging results of the TR-TR differential probe. The defect is actually located between the red area and the blue area in the figure, and the actual length of the defect is smaller than the imaging area. TR-TR differential probes cannot directly reflect the size, shape and location of defects. In this paper, a defect feature extraction algorithm is proposed based on the analysis of point spread function (PSF) of the probe.

**Figure 23:**
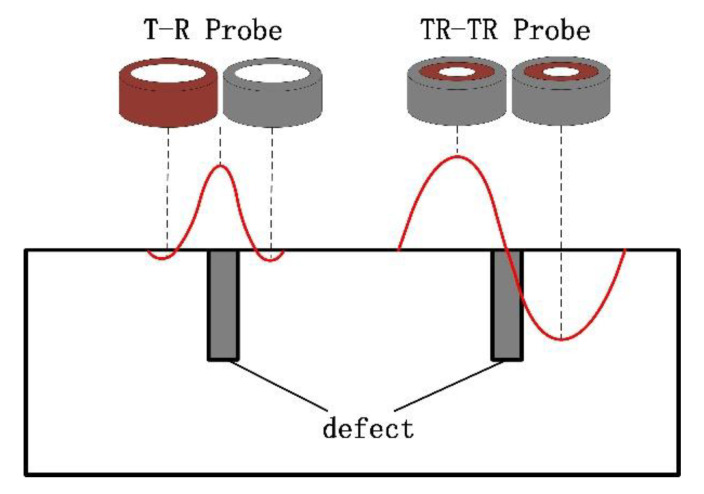
Comparison of PSF of traditional T-R probe and new differential probe.

The PSF in eddy-current testing refers to the variation in the measured voltage when the probe scans a small surface defect on the sample. The PSF is an inherent property of the probe and is related to the spatial location and size of the coil. Figure 23 shows a schematic diagram of the PSF of the two probes used in this experiment. It can be seen from the figure that the T-R probe forms an obvious wave peak at the defect position, and the defect position information can be accurately displayed during imaging. The PSF of the differential probe forms a crest and a trough on both sides of the defect, while the measured voltage change at the centre of the defect is 0, forming a blind spot.

When the TR-TR probe scans at a constant speed, the form of the PSF is shown in Formula (3). Here, *t* = 0 corresponds to the moment when the probe moves to the centre of the defect, and w is the time it takes for the probe to reach the defect from one end until the other end of the probe leaves the defect. *f*(*t*) is the PSF value corresponding to time *t*.

(3) $$F\left(t\right)=\{\begin{array}{cc} -f\left(t\right) & -0.5w<t<0\\ 0 & t=0\\ f\left(t\right) & 0<t<0.5w \end{array}$$

According to the above analysis, it is difficult for the new differential probe to locate defects directly in imaging, and the defect information is implied in *F*(*t*). In this paper, a defect feature extraction algorithm for differential probes is proposed. The algorithm extracts the defect information by intercepting the part of the PSF that can reflect the defect information as the convolution kernel and convolving the convolution kernel and the original detection signal. It works as follows. In the convolution formula shown in Formula (4), v(t−a) is the original detection signal, and *F*(*a*) is the convolution kernel selected based on the PSF. Convolution operations are performed on v(t−a) and *F*(*a*) to obtain the result *S*(*t*) after defect feature extraction.

(4) $$S\left(t\right)=\overset{a=0.5w}{\underset{a=-0.5w}{\sum }}F\left(a\right)v\left(t-a\right)$$

The original detection signal can be expressed by Formula (5):

(5) $$v\left(t\right)=\{\begin{array}{cc} \mathrm{Bias}+\mathrm{noise}\left(t\right); & t<0.5w\;or\;t>0.5w\\ \mathrm{Bias}+\mathrm{noise}\left(t\right)+F_{1}\left(t\right); & -0.5w<t<0.5w \end{array}$$

When the probe is at the defect-free position, the detection signal consists of residual voltage bias caused by the incomplete symmetry of the two sets of coaxial coils and noise signal noise (*t*) caused by lift-off fluctuations. The residual voltage bias is a constant, and the noise signal (*t*) is a small random value. At the defect position, in addition to these two items, there is an additional item of the induced voltage variation value $F_{1}\left(t\right)$ caused by the defect.

The primary problem of this algorithm is the selection of the convolution kernel. For the TR-TR differential probe, the blind spot position corresponds to the central area of the defect, so the selection of the convolution kernel should take the blind spot as the centre and extend a certain distance equidistantly to both sides. Assuming that the width of the selected convolution kernel is *n* times the width of the PSF (0 < *n* < 1), the defect feature extraction result is

(6) $$S\left(t\right)=\{\begin{array}{cc} 0+\overset{a=nw}{\underset{a=-nw}{\sum }}F\left(a\right)noise\left(t-a\right) & t<-0.5w\;or\;t>0.5w\\ \overset{a=nw}{\underset{a=-nw}{\sum }}F\left(a\right)F_{1}\left(t-a\right)+\overset{a=nw}{\underset{a=-nw}{\sum }}F\left(a\right)noise\left(t-a\right) & -0.5w<t<0.5w \end{array}$$

since the noise signal noise (*t*) is much smaller than the bias signal bias and the defect signal $F_{1}\left(t\right)$, the influence of the noise signal noise (*t*) on the defect feature extraction can be ignored. Thus, the defect feature extraction result becomes

(7) $$S\left(t\right)=\{\begin{array}{cc} 0 & t<-0.5w\;or\;t>0.5w\\ \overset{a=nw}{\underset{a=-nw}{\sum }}F\left(a\right)F_{1}\left(t-a\right) & -0.5w<t<0.5w \end{array}$$

**Figure 24:**
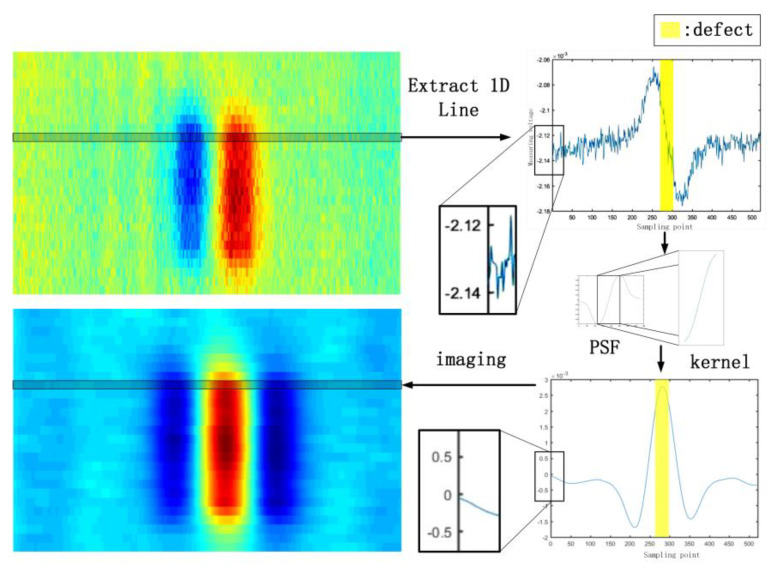
Flow chart of defect feature extraction algorithm based on point spread function.

According to Formula (7), after the original signal is processed by the defect feature extraction algorithm, the residual voltage caused by the incomplete symmetry of the two sets of concentric coils can be eliminated, and the defect information is more prominent. The specific processing process of the feature extraction algorithm in this paper is as follows. As shown in Figure 24, first, the PSF of the new probe is obtained through simulation, and the amplitude of the PSF is scaled to the interval (−1, 1). The point expansion function is interpolated so that the number of convolution kernel data points after interpolation is basically the same as the number of sampling points in the same moving range of the device. The part of the PSF that can reflect the defect information is selected as the convolution kernel. Feature extraction is performed on the original one-dimensional measurement signal, and the feature extraction result is reimaged.

**Figure 25:**
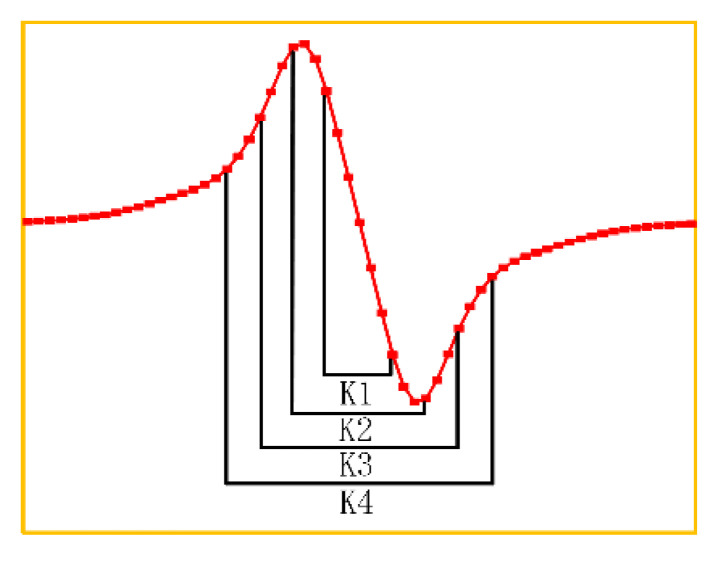
TR-TR differential probe PSF.

In this paper, multiple convolution kernels are selected based on the PSF, and the selection strategy of the final convolution kernel is determined by comparing the imaging effects. The PSF of the coil used in the experiment obtained by simulation is shown in Figure 25. Different regions in the PSF are intercepted as convolution kernels, and K1 to K4 are obtained.

**Figure 26:**
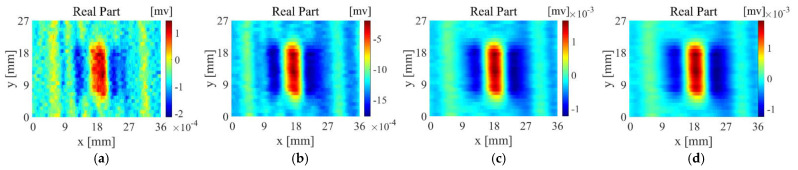
Feature extraction results of sample 3: (**a**) K1; (**b**) K2; (**c**) K3; (**d**) K4.

Taking sample 3 as an example, Figure 26 shows the result of feature extraction on the measurement results of sample 3. From the figure, it can be seen that the defect position can be basically located after feature extraction by K1, but the contrast between defect and the background region is low. The feature extraction results from K3 to K4 are basically the same. The dark red area is closer to the actual position and size of the defect, but the dark blue area on both sides of the defect that cannot reflect the defect features is wider and has more redundant information. However, in addition to extracting defect information, K2 can also extract the weaving structure of the background area. In summary, the convolution kernel of the feature extraction algorithm is selected as K2.

**Figure 27:**
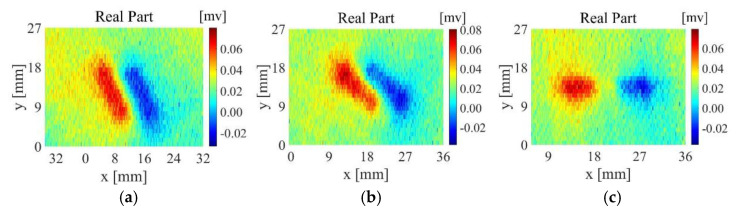
Direct imaging of sample 3 at different scanning angles: (**a**) the angle between the probe and the length of the defect is 30°; (**b**) the angle between the probe and the length of the defect is 60°; (**c**) the angle between the probe and the length of the defect is 90°.

**Figure 28:**
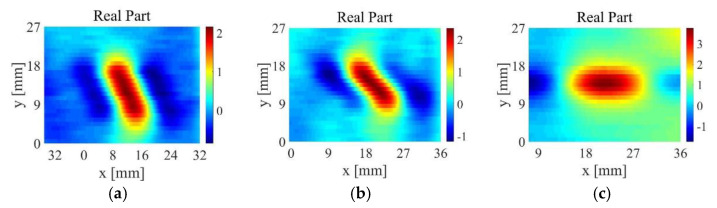
Feature extraction results of sample 3: (**a**) the angle between the probe and the length of the defect is 30°; (**b**) the angle between the probe and the length of the defect is 60°; (**c**) the angle between the probe and the length of the defect is 90°.

Figure 26 is an image of the TR-TR differential probe scanning the sample 3 along the direction perpendicular to the length of the defect. In order to verify the robustness of the algorithm in this paper, the TR-TR probe scans the sample 3 from multiple angles, and then processes the original detection signal through the feature extraction algorithm in this paper. The angle between the TR-TR differential probe and the defect length is changed in 30° steps, and the obtained original image is shown in Figure 27. The images after feature extraction are shown in Figure 28. 

As shown in Figure 28, the algorithm proposed in this paper can basically reflect the size and shape of defects. The experimental results can show that the algorithm proposed in this paper has a wide range of applicability.

**Figure 29:**
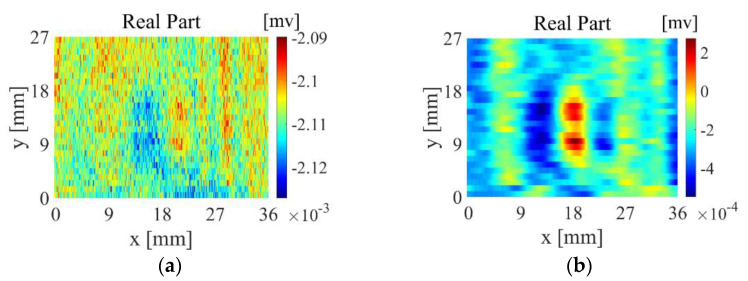
Detection results of small defects: (**a**) direct imaging; (**b**) imaging based on feature extraction algorithm.

For smaller defects, the weak measurement signal is more susceptible to noise interference, and the defect information is not obvious in the imaging results pre-processing. Figure 29a shows the direct imaging result. The contrast between the defect part and the background area is not high, which easily causes missed inspection in the actual inspection process. Figure 29b shows the measurement results of the new differential probe on the rectangular defect of 4 mm × 0.4 m × 0.4 mm on the CFRP sample with the same structure as Sample 3 after applying the feature extraction algorithm. It can be seen from the figure that the contrast between the defect part and the surrounding area is higher in the imaging result, the defect can be clearly observed, and the location of the defect is clear.

**Figure 30:**
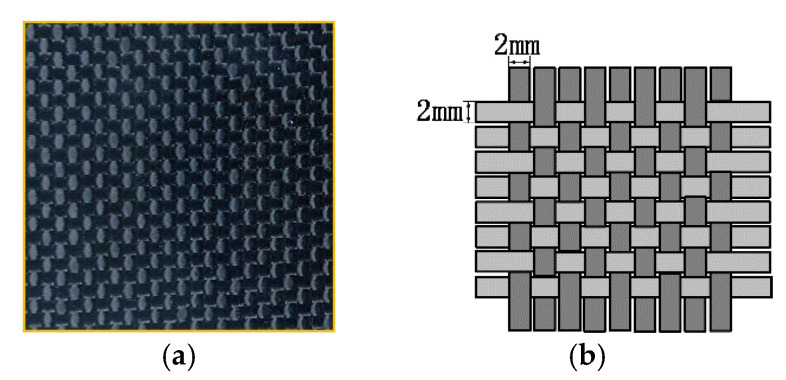
(**a**) Photo of braided structure CFRP; (**b**) schematic diagram of braided structure.

**Figure 31:**
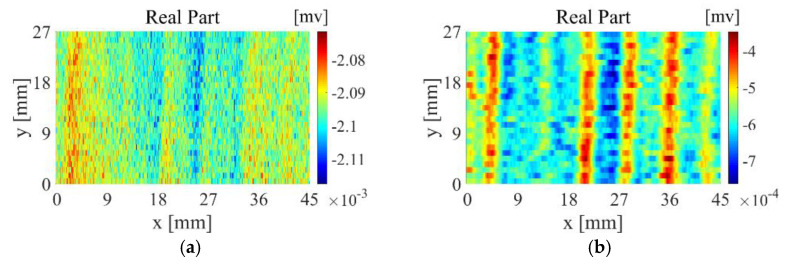
CFRP images of braided structures: (**a**) original image; (**b**) image after feature extraction.

As shown in Figure 30, the CFRP of the braided structure is composed of interwoven carbon fibre bundles, and voids are generated between adjacent fibre bundles during weaving. Theoretically, the quality of the CFRP weave can be evaluated by detecting the voids between fibre bundles through eddy-current detection methods. However, the new differential probe used in the experiment is large in size and low in spatial resolution, making it difficult to detect tiny gaps between adjacent fibre bundles. Figure 31a shows the imaging results of the TR-TR differential probe scanning the braided CFRP sample. From the figure, only the fibre arrangement direction can be roughly observed, but the fibre texture can hardly be observed. As shown in Figure 31b, after feature extraction, the arrangement direction and texture of fibres can be clearly observed.

## 6. Conclusions

This paper has proposed a new differential TR-TR probe for CFRP inspection. Compared with the traditional T-R probe, the new design has less sensitivity to scanning angle, increased sensitivity to defect and better image SNR. The performance has been verified both in FEM simulation and through experimental tests on orthogonally laminated CFRP and braided CFRP. In addition, to improve the defect localization using the TR-TR differential probe, a defect feature extraction algorithm was proposed, and the selection strategy of the convolution kernel was discussed. The results showed that the imaging results processed by the feature extraction algorithm are more accurate for defect location with a higher contrast between the defect area and the background area.

## Figures and Tables

**Table 1:** Coil parameters of simulation model.

Model Variables	Inner Diameter	Outer Diameter	Turns	Height
Tx Coil	2 mm	3 mm	150	6 mm
Rx Coil	3.6 mm	4.6 mm	150	6 mm

## Data Availability

Not applicable.
